# A Role for Exocytosis in the Spatial Regulationof Actin Organization

**DOI:** 10.1100/tsw.2003.119

**Published:** 2003-12-18

**Authors:** Xiang-Dong Gao, Stefan Albert

**Affiliations:** Department of Cell and Developmental Biology, University of Pennsylvania, 421 Curie Boulevard, Philadelphia, PA 19104-6058, USA

**Keywords:** cell polarization, exocytosis, secretion, Msb3p, Msb4p, Cdc42p, Sec4p, actin cables, actin patches, Rab GTPase, Rab GAP, membrane trafficking, endocytosis, yeast

## Abstract

The efficient organization of the actin cytoskeleton is important for many cellular functions. However, how the local actin organization is regulated in a cell is not well understood. By using yeast mutants defective in actin organization and secretion, we demonstrated that exocytosis plays a role in the spatial regulation of actin organization. Our findings suggest that the actin cytoskeleton, exocytosis, and perhaps endocytosis, may depend on each other for efficiency and reinforce each other.

Cell polarization is critical for diverse cellular and developmental processes in many organisms[1]. Defects for a cell to polarize correctly could lead to human diseases including the Wiskott-Aldrich syndrome. In the budding yeast *Saccharomyces cerevisiae*, the generation of a new bud is a highly organized cell polarization process that involves several hierarchical and interdependent events, including bud-site selection, actin and septin organization, polarized secretion, and organelle inheritance[2].

Cdc42p, a Rho-family GTPase, is required for organizing actin cables and actin patches at the presumptive bud site[3]. To understand how Cdc42p accomplishes this function, a genetic screening for multicopy suppressors was carried out in a yeast *cdc24* mutant, which is defective in the activation of Cdc42p. The *MSB3* gene was isolated among several suppressors from this screening[4]. Multicopy overexpression of *MSB3* suppressed the budding and actin organization defects of several *cdc42* mutants, whereas double deletion of *MSB3* and its close homologue, *MSB4*, displayed synthetic lethality with several *cdc42* mutants[4,5]. Surprisingly, the encoded proteins, Msb3p and Msb4p, possess a conserved TBC domain (also called GYP domain) shared among many Rab GTPase-activating proteins (GAP)[6,7], and indeed, both proteins displayed GAP activities toward several Rab GTPases *in vitro*[6,7]. These findings suggest that Msb3p and Msb4p are involved in promoting polarized growth and perhaps polarized actin organization. In a recent study published in *The Journal of Cell Biology*[5], we established a physiological function of Msb3p and Msb4p in promoting efficient exocytosis and actin organization, and furthermore, we established an important link connecting exocytosis to the spatial organization of the actin cytoskeleton.

The actin cytoskeleton, which includes actin cables and actin patches, plays an essential role in the budding process. During bud emergence, actin cables are oriented along the mother-bud axis and are believed to direct the transport of secretory vesicles, *ASH1* mRNA, and organelles into the growing bud[8]. Meanwhile, actin patches are concentrated in the growing bud and are thought to mediate endocytosis[9]. Yeast mutants that are either devoid of actin cables, such as the conditional tropomyosin *tpm1-2 tpm2*Δ mutant[10] and the formin *bni1 bnr1*Δ mutant[11,12], or are unable to organize actin cables and patches in a polarized fashion, such as conditional *cdc42-1* mutant[3], cannot generate a bud due to a failure to polarize cell growth to the presumptive bud site. As a result, they grow isotropically and become large and round. So far, it is not well understood how the spatial organization of actin cables and patches is achieved. Actin patches are thought to consist of short actin filaments nucleated by the Arp2/3 complex[13,14]. In mammalian cells, activated Cdc42 activates WASP through direct binding, which in turn activates the Arp2/3 complex[15,16]. However, the WASP homolog in the budding yeast, Las17p/Bee1p, does not bind Cdc42p directly[17]. So it is still an open question whether Cdc42p plays a direct role in the organization of actin patches. One report suggests that Cdc42p may be involved in the activation of the Arp2/3 complex indirectly through its downstream effectors[17]. By contrast, Cdc42p appears to have a more defined role in the organization of actin cables[18]. Cdc42p is thought to function by recruiting Bni1p (formin) to the incipient bud site[19], and perhaps is also involved in activating Bni1p's actin-nucleating activity[20,21] that is required for the formation of actin cables[11,12]. Our study[5] and another study published recently[22] both indicate that Cdc42p function and actin organization are modulated by exocytosis. The latter study further showed that Cdc42p was delivered to the polarity sites on secretory vesicles along actin cables, and thus provides a mechanism for the involvement of exocytosis in these processes. These findings are significant since they provide a novel view on how the organization of the actin cytoskeleton is spatially regulated.

By using a combination of genetic, cytological, and biochemical methods, we demonstrated that both Msb3p and Msb4p function as GAPs for Sec4p, an essential Rab GTPase that acts during the transport of secretory vesicles from the Golgi to the growing bud, and also the docking/tethering of these vesicles on the plasma membrane[23]. Since Sec4p and Cdc42p function in distinct pathways, our findings raised an intriguing question: Could exocytosis normally play a role in actin organization? Several lines of evidence support this hypothesis. First, the ability of multicopy *MSB3* to suppress the budding and actin-organization defects of *cdc42* mutants requires its GAP activity. Second, the GAP activity of Msb3p and Msb4p is required for the efficient organization of actin patches. Third, the disassembly of actin cables in *tpm1-2 tpm2*Δ cells causes a gradual depolarization of actin patches into the mother cell[10]. However, a blocking of the fusion of secretory vesicles with the plasma membrane in *tpm1-2 tpm2*Δ cells prevents the depolarization of actin patches, although these actin patches in the bud are still short lived.

Our findings that exocytosis modulates the efficiency of Cdc42p function and actin organization could be explained by assuming that actin-assembly activators are normally carried into the growing bud on secretory vesicles, and these factors are deposited on the plasma membrane through vesicle fusion. The local increase in the concentration of these factors on the plasma membrane could somehow lead to the assembly of new actin patches and cables. So far, very little is known about the identity of these factors and how they control the assembly of actin filaments. Some possible candidates could be Cdc42p[17,22], Rho1p[24], or Bud6p[25]. This model could explain some of the genetic interactions we observed between *MSB3*, *MSB4,* and *CDC42*. The synthetic lethality between *msb3Δ msb4Δ* and several *cdc24* and *cdc42* alleles could result from a failure to deliver enough Cdc42p molecules to generate a pool of active GTP-bound Cdc42p at the incipient bud site. Overexpression of Msb3p in *cdc42* mutants, on the other hand, could help cells to generate such an active Cdc42p pool through promoting efficient delivery of Cdc42p to the incipient bud site, and thus facilitate actin organization.

Our model is also consistent with the results in a recent report on the Cdc42p$^{Q61L}$-mediated spontaneous cell polarization[22]. In this report, the authors proposed a positive feedback loop that involves stochastic increase of local activated Cdc42p on the plasma membrane, followed by actin polymerization activated by GTP-bound Cdc42p, and more Cdc42p delivery to this site through actin cable-directed vesicular transport and fusion machinery. This process could generate a polarity axis, and thus, drive cell polarization[22]. Both models could explain the initial establishment of cell asymmetry, but could not explain how such a polarity axis can be sustained at a steady level, and how the polarity axis may disassemble when a cell switches its growth to a different site during cytokinesis, or enters an isotropic growth phase. For this aim, we believe that some forms of down-regulation mechanism that destabilizes the polarity axis may exist to prevent the cell from approaching a no-return situation. Such a negative regulation could be provided by actin patches–mediated endocytosis[9], which may serve as a practical means to eliminate polarity proteins including Cdc42p from the polarity sites. Therefore, we propose that a combination of both positive and negative feedback loops will be necessary to achieve such goal. This would involve the delivery of Cdc42p along actin cables to the presumptive bud site, and followed by the assembly of actin patches at this site, and subsequent Cdc42p internalization through endocytosis.

In summary, our study indicates that exocytosis modulates Cdc42p function on which the organization of actin cables depends, and in contrast to a widely accepted view that actin cables direct exocytosis, we revealed a novel role of exocytosis in the organization of actin patches, which has been underappreciated before. Considering that endocytosis normally helps recycling of v-SNAREs and lipids for further rounds of exocytosis, these complex interactions between actin cables, actin patches, exocytosis, and endocytosis may eventually provide both positive and negative regulations to each other, and all these regulations are indispensable for a cell to accomplish normal morphogenesis.

## ACKNOWLEDGMENTS

Original work by the authors was done in Dr. Erfei Bi's laboratory at University of Pennsylvania, and in Dr. Dieter Gallwitz's laboratory at Max Planck Institute for Biophysical Chemistry, Germany, and was supported by National Institutes of Health grant GM59216 to Dr. Erfei Bi, and grants to Dr. Dieter Gallwitz from the Deutsche Forschungsgemeinschaft and Fonds der Chemischen Industrie and Human Frontier Science Program. We thank all the laboratory members for their support and encouragement throughout this work.

## BIOSKETCHES

**Xiang-Dong Gao** is a Research Associate in the Department of Cell and Developmental Biology at the University of Pennsylvania. He received a Ph.D. in Genetics from Fudan University, China.

**Štefan Albert** is an Investigator at the Institute of Medical Radiation and Cell Research, University of Würzburg, Germany.
